# Complete chloroplast genome of *Dendrobium thyrsiflorum* (Orchidaceae)

**DOI:** 10.1080/23802359.2019.1667918

**Published:** 2019-09-23

**Authors:** Yun-Yun Pan, Ting-Zhang Li, Jian-Bing Chen, Jie Huang, Wen-Hui Rao

**Affiliations:** a Key Laboratory of National Forestry and Grassland Administration for Orchid Conservation and Utilization, Shenzhen, China;; b Shenzhen KeyLaboratory for Orchid Conservation and Utilization, The Orchid Conservation and Research Centre of Shenzhen, The National Orchid Conservation Centre of China, Shenzhen, China

**Keywords:** *Dendrobium thyrsiflorum*, chloroplast genome, phylogeny, Orchidaceae

## Abstract

*Dendrobium thyrsiflorum* H. G. Reichenbach ex André is an endemic herb with ornamental and medicinal orchid value distributed in Southeast of Yunnan of China. Here, we report and characterize the complete chloroplast (cp) genome sequence of *D. thyrsiflorum* in order to provide genomic resources helpful for its identification, conservation and utilization. The complete cp genome of *D. thyrsiflorum* is 160,123 bp, including one large single-copy region (LSC, 88,001), one small single-copy region (SSC, 21,142), and two inverted repeat regions (IRs, 25,490). The cp genome contains 143 genes, consisting of 110 unique genes (80 protein-coding genes, 26 tRNAs, and 4 rRNAS). The phlyogenetic relationships show that *D. thyrsiflorum* is closely related to other species of *Dendrobium*.

*Dendrobium thyrsiflorum* H. G. Reichenbach ex André is a plant of the family Orchidaceae. The genus *Dendrobium* is established by Swartz in 1799, and now the recognized species are about 78 species (14 endemic) in China (Chen et al. 2009). Some new species of this genus have been published in recent years (Xu et al. 2014; Leonia et al. 2015; Phattaravee et al. 2017; Tian et al. 2017). Many species of this genus have high medicinal value with a long medicinal used history in china. The main chemical components of *Dendrobium* are polysaccharides, alkaloids with anti-cataract, lowering blood sugar, anti-tumor, and anti-oxidation effects (Bao 2007; Lic 2012; Zhang et al. 2012).

In this study, leaf samples of *D. thyrsiflorum* were obtained from the Orchid Conservation and Research Centre of Shenzhen. The voucher specimens (Z.J. Liu 3431) were deposited in the herbarium of National Orchid Conservation Center. The total genomic DNA was extracted from fresh leaf by using the modified CTAB procedure of Doyle and Doyle (1987), and sequenced on Illumina Hiseq 2500 platform (Illumina, San Diego, CA). Genome sequences were screened out and assembled with MITObim v1.8 (Hahn et al. 2013), which resulted in a complete circular sequence.

The cp genome sequence of *D. thyrsiflorum* (GenBank accession MN306203) is 160,123 bp in length, and includes one large single-copy region (LSC, 88,001), one small single-copy region (SSC, 21,142), and two inverted repeat regions (IRs, 25,490). And it encoded 143 genes, of which 110 were unique genes (80 protein-coding genes, 26 tRNAs, and 4 rRNAS). The overall GC content is 37.1%.

To confirm the phylogenetic position of *D. thyrsiflorum* and analysis the phylogenetic of *Dendrobium*, a molecular phylogenetic tree was constructed based on the maximum-likelihood (ML) methods with forty published complete cp genome sequences of *Dendrobium* and two *Pleione* species as outgroup. The ML analysis was performed using the CIPRES Science Gateway web server (RAxML-HPC2 on XSEDE 8.2.10) with 1000 bootstrap replicates and settings as described by Stamatakis et al. (2008). Results showed that *D. thyrsiflorum* is mostly related to other species of *Dendrobium* (Figure 1). This newly reported chloroplast genome provides a good foundation for the identification and genotyping of *Dendrobium* species, and further promote the basis for the protection of germplasm resources and the breeding of new varieties.

**Figure 1.**
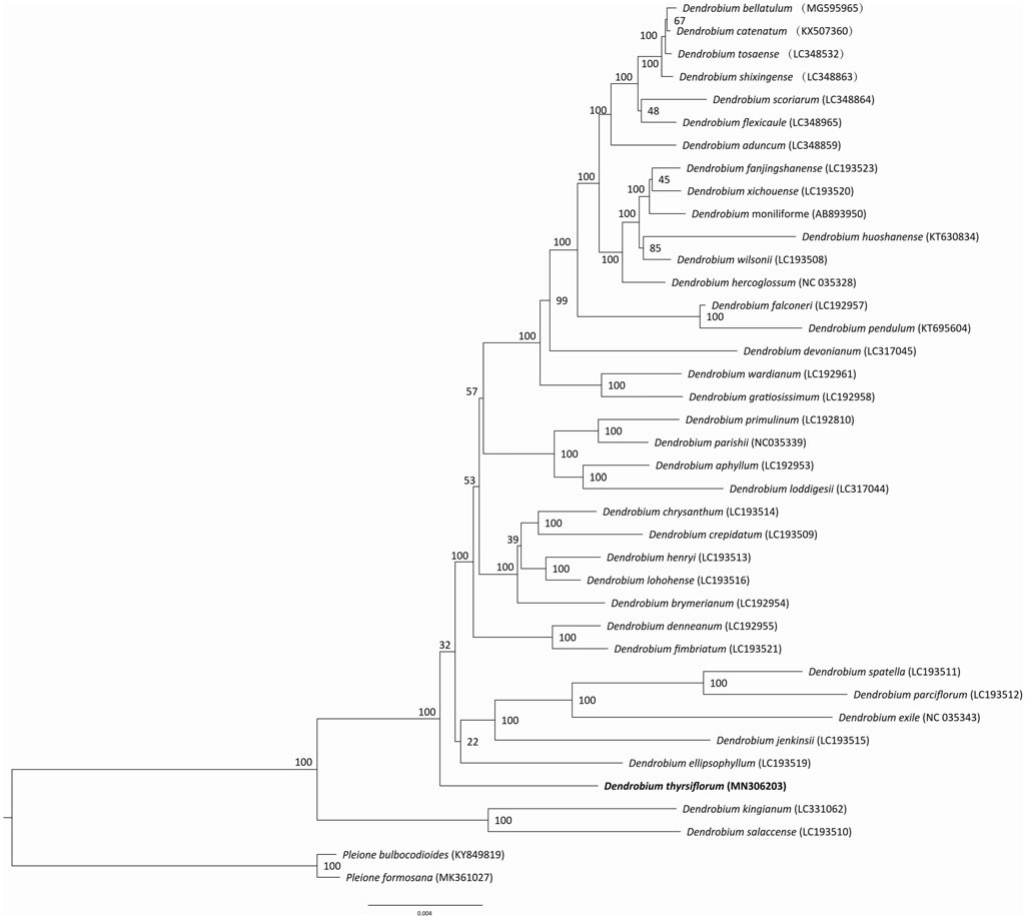
Phylogenetic position of *Dendrobium thyrsiflorum* inferred by maximum likelihood (ML) of complete cp genome. The bootstrap values are shown next to the nodes.
